# The effects of an educational program on depression literacy and stigma among students of secondary schools in Jazan city, 2016: A cluster-randomized controlled trial study protocol

**DOI:** 10.1097/MD.0000000000009433

**Published:** 2018-05-04

**Authors:** Hussain Darraj, Mohamed Salih Mahfouz, Rashad Al Sanosi, Mohammed Badedi, Abdullah Sabai

**Affiliations:** a Jazan Health Affairs, Ministry of Health; b Family and Community Medicine Department, Faculty of Medicine; c Substance Abuse Research Center (SARC), Jazan University, Jazan; d Public Health Administration, Jazan Health Affairs, Ministry of Health, Saudi Arabia.

**Keywords:** depression, Jizan, literacy, stigma

## Abstract

**Background:**

Depression is a serious mental health disorder and characterized by sadness, loss of interest in activities, and decreased energy. The aim of this study is to assess the effectiveness of the school intervention program on depression literacy and stigma among students of secondary schools.

**Design and methods:**

A cluster randomized trial will be conducted on sample of 360 students to assess the depression literacy and stigma towards depression before and after a designed intervention educational program. The intervention consists of a package of 2 lectures, 1 video contact, and group discussion of 5 myths about depression, posters, and brochure. The target population consists of all secondary school students in Jazan, where there are 13 secondary schools will be stratified according to sex (6 schools for boys and 7 schools for girls).

**Expected impact for public health:**

The results of the study will provide evidence of the efficacy of educational intervention programs on increasing depression literacy among students of secondary schools in Jazan City. The expected outcome of this study is to increase the depression literacy rate among high school students in the intervention group.

## Abstract 2

Supplemental Digital Content is available in the text

## 1 Introduction

Depression is a serious mental health disorder. It is generally characterized by sadness, loss of interest in activities, and decreased energy.^[1]^ Evidence suggests that the onset of major depression predominantly manifests during adolescence. Furthermore, it influences a child's psychological, social, and academic functioning. Due to this, they will be vulnerable to the risk of substance abuse and suicidal behavior.^[2,3]^ In developing countries, depressive disorders represent a key determinant of health-related disability, and they are considered a major source of nonfatal disease in developed countries.^[4,5]^ Globally, it is estimated that almost 350 million people suffer from depression.^[6]^

In Saudi Arabia, a study has shown that the prevalence of depression among male secondary school students in Abha City is 38.2%.^[7]^ Another study done in the same region found depression in 13.9% of female students.^[8]^ A study done in Al-Ahsa found the symptoms of depression in 24.4% of King Faisal University students. Suicidal ideation in the past 4 weeks was reported by 1.1% of students. Major depression was significantly higher among females.^[9]^ Another study found that the prevalence of depression was 22.4% as moderate, 7.3% as severe, and 3.7% as very severe, with depression occurring 1.5 times more often in girls than in boys.^[10]^

Considerable amount of the current literature pays particular attention to the importance of educational intervention for depression literacy.^[11]^ Rahman et al^[12]^ argued that the school programs succeeded in improving awareness of mental health in school children and the community. The school children were receptive to the program, and shared their new understanding with family, friends, and neighbors. An Internet-based intervention study conducted in Australia suggests that brief mental health literacy and destigmatization improves knowledge and may decrease stigma but does not increase help-seeking for young elite athletes.^[13]^ Another study conducted in Sweden suggested that 42.7% of the respondents identified depression. Depression was recognized more often by women than men. Professional help was suggested by a minority of the respondents for managing symptoms of depression.^[14]^ Overall, these studies highlight the need for educational intervention about depression literacy at the school level. As far as we are aware, there is no similar study conducted in Jazan area that deals with depression.

The main objective of this study is to measure the effect of an educational intervention program about depression on depression literacy and stigma among secondary schools in Jazan City, during the academic year of 2016.

## 2 Methods and analysis

### 2.1 Trail design, study area, and target population

A cluster-randomized trial design will be employed for this study. The study will be conducted in Jazan City, the capital of Jazan region, lies in the south-western part of the Kingdom of Saudi Arabia. There are 13 governmental secondary schools (6 for boys and 7 for girls) in Jazan City. Inclusion criteria involves; students who are enrolled in one of the selected schools; participants in the age group of 10 to 19 years, and students who agreed to participate in the study (consent will be obtained from students, and proxy consent will be obtained from guardians). There were no exclusion criteria for participation in this study.

### 2.2 Sampling size and design

The sample size calculation will be conducted to determine the number of participants required. Based on the study's design, an intracluster correlation coefficient (ICC) of 0.02 will be used in sample size calculation based on similar studies,^[15,16]^ and an effect size of 0.5 will be used to detect the medium effect of the intervention based on similar studies by Perry et al^[15]^ and Gulliver et al,^[13]^ which used the same design and assessment tool (the depression literacy questionnaire [D-Lit]). Other parameters for estimating sample size include a 95% confidence interval and an Alpha coefficient of 5%. Accounting for a 10% non-response and/or drop-out rate, the total required sample size will be 360 students, 180 of whom will be recruited for the intervention group and 180 for the control group. The unadjusted sample size calculation will be conducted using the G∗Power program (version 3.1.9.1; Heinrich Heine Universität, Düsseldorf, Germany)^[17]^, then adjusted for the cluster design effect according to the methods outlined in Campbell et al.^[18]^

For sampling procedures in stage one 13 secondary schools in Jazan city will be stratified according to sex (6 schools for boys and 7 schools for girls). In stage two, 1 school from each stratum will be selected at randomly for the control group, and another one from each stratum will be selected at randomly for the intervention group using the closed envelope technique. Finally, in sage three and based on the calculated sample size of 360, random sampling technique will be used to recruit 180 students from the control schools (1 boy's school and 1 girl's school) and 180 students from the intervention schools (1 boy's school and 1 girl's school) (see Figure 1, Supplemental Content which illustrates the sampling procedures).

### 2.3 Data collection instruments and trail phases

#### 2.3.1 Phase one

Pre-intervention assessment through the D-Lit, the depression stigma scale (DSS), and the self-stigma depression scale (SSDS) will be carried out for the control and intervention groups to establish a baseline information. Participants will be asked to provide basic demographic information (sex, age, grade, school level, residence, and nationality).

The D-Lit assesses mental health literacy specific to depression. The instrument was developed by the National Institution for Mental Health Research in Australia, lead developer Kathy Griffiths. The D-Lit questionnaire's Cronbach alpha is 0.70, and its test–retest reliability is *r* = 0.71.^[13]^ The questionnaire consists of 22 true-or-false items. Respondents can answer each item with 1 of 3 options: “true,” “false,” or “don’t know.” Each correct response receives 1 point. Higher scores indicate higher mental health literacy of depression. D-Lit items are about depression symptoms, management, treatment, and duration, as well as differentiation between depression and other mental illnesses.

The DSS is designed to measure stigmas associated with depression. The scale was developed by the National Institution for Mental Health Research in Australia, lead developer Kathy Griffiths. The DSS has 2 subscales, which measure 2 different types of stigma: personal and perceived. The Personal Stigma Subscale measures stigma in the respondents’ own attitudes towards depression by asking them to indicate how strongly they personally agree with 9 statements about depression. The Perceived Stigma Subscale measures the respondent's perception about the attitudes of others towards depression by asking them to indicate what they think most other people believe about the same 9 statements. Responses to each item are measured on a 5-point scale (ranging from 0, “strongly disagree” to 4, “strongly agree”). Higher scores indicate higher levels of depression stigma. Cronbach alpha values for the total, personal, and perceived depression stigma scales were 0.78, 0.76, and 0.82, respectively.^[19]^

The SSDS is designed to assess the extent to which a person holds stigmatizing attitudes towards themselves in relation to having depression. The scale was developed by the National Institution for Mental Health Research in Australia, lead developer Lisa Barney. The SSDS is a 16-item scale with 4 subscales: shame, self-blame, social inadequacy, and help-seeking inhibition. Responses to the self-stigma items are measured on a 5-point scale (ranging from 1, “strongly agree,” to 5, “strongly disagree”). Higher score indicates greater self-stigma. Cronbach alpha values for the SSDS total, shame subscale, self-blame subscale, help-seeking inhibition subscale, and social inadequacy subscale are 0.87, 0.83, 0.78, 0.79, and 0.79, respectively.^[20]^

#### 2.3.2 Phase two: intervention

The intervention program will be held in a school setting and supervised by a team consisting of a psychiatrist, a public health physician, and a social worker. The program will be delivered by a teacher from each school after they have received short training to ensure uniformity of the interventions. The educational material will consist of written material created by a specialized team (a psychiatrist, a public health physician, and a social worker). It will be designed according to educational and contact strategies to increase depression literacy and to reduce stigma toward depression for secondary school students and based on studies of mental illness stigma reduction theory.^[21]^ The video, lectures, and group discussion will be presented in equipped classrooms in each intervention school. The intervention will be given over a period of 2 weeks as follows and the educational strategy will be:

##### 2.3.2.1 Lectures

There will be 2 lectures of around 30 minutes; 1 lecture will be held each weak. Thirty students will attend each lecture. Therefore, each intervention school (90 students) will be divided into 3 groups (30 students in each); the first lecture will cover recognition, risk factors, and causes of depression. The second lecture will cover depression treatments—including antidepressants and cognitive behavioral therapy—as well as the professional help available and appropriate ways to seek help.

##### 2.3.2.2 Group discussion

Each lecture will be followed by a group discussion concerning common myths about depression. After the lecture, for 5 minutes for each myth presented, the students will be divided into groups of 10 for discussion. Myths will be displayed on the monitor, and students will be asked to talk about their opinions, specifically whether they agree or disagree with the myths. After 5 minutes, the teacher will give them the correction for the displayed myth.

##### 2.3.2.3 Brochures

A printed brochure will be structured to include several components, including how to recognize depression, risk factors, and causes, treatments for depression—including antidepressants and cognitive behavioral therapy—the professional help available and appropriate help-seeking behaviors. The brochures will be distributed after the second lecture. Students will then be asked to read these brochures for 10 minutes, and they will be encouraged to ask questions about the material.

##### 2.3.2.4 Posters

Four posters with mental-health slogans about depression will be distributed around the school in visible places. Poster themes will include the following: “Get support from your doctor, family, friends, and school,” “An untreated mental disorder can lead to more severe and difficult to treat illness,” “If you are worried, sad, angry, or just don’t feel ok, talk to someone about how you feel. Reach out for help. Don’t be afraid,” and “Getting help shares the burden. Believing in yourself makes your load lighter.”

##### 2.3.2.5 Contact strategies

Numerous studies support the role of contact with an individual who has personal experience with mental illness in helping to reduce stigma.^[22,23–25]^ Research suggests that video contact can work as effectively as a live presentation.^[26,27]^ Thus, we will show a video of a young man who has been diagnosed (i.e., not an actor) with depression. The video will present information that is moderately disconfirming of prior stereotypes by balancing presentation of the individual's difficulties resulting from depression with his ability to live a normal life.

#### 2.3.3 Phase three

Post-intervention assessment by the D-Lit, DSS, and SSDS questionnaires will be conducted for the control and intervention groups immediately after the intervention and during a follow-up after 3 and 12-months.

### 2.4 Statistical analysis and data management plan

The main analysis will include the data collected from all respondents. Differences between socio-demographic variables will be examined using chi-squared analyses. The Statistical Package for the Social Sciences (SPSS) (version 20) will be used to analyze the data. Differences between mean scores of D-Lit, DSS, and SSDS for the intervention and control groups—as well as changes in mean scores over time for each group—will be tested with either a paired *t* test or with Welch *t* test when the variances of the 2 comparison groups are not equal. To examine differences between groups at the baseline, Student *t* test—or the Mann–Whitney *U* test if appropriate—will be performed for pre-intervention D-Lit, DSS, and SSDS scores. All tests will be adjusted for cluster design using the design effect.^[28]^*P*-values <.05 will be used in all analyses.

### 2.5 Expected outcome

The expected outcome of this study is an increase in depression literacy among secondary school students in the intervention group.

### 2.6 Ethical consideration

This study protocol was approved by a Faculty of Medicine Research Ethics Committee at Jazan University. Consent will be received from the managers of the selected schools. Before participation, students will be informed that the collected information will be kept anonymous and that participation is absolutely voluntary. The participants will be assured that they could withdraw from the study at any time. Informed consent will be sought from the eligible participants following a full disclosure regarding the study. Proxy consent for the children will be obtained from their guardians and from each child who is eligible to participate in the study before randomization.

### 2.7 Protocol amendment and data dissemination

The results of Trial will be disseminated through scientific conference and seminars presentations and by publication in peer reviewed scientific journals. Modifications of the trial protocol will only be done in consultation with investigators. Substantial amendments of the protocol require the approval of the Ethics Committee.

## 3 Discussion

Many studies have concluded that educational intervention centered around the facts about depression and destigmatization improves knowledge and may decrease stigma.^[29]^

Accumulated evidences suggested the importance of educating adolescents about depression, because this is the peak period for the onset of mental disorders, with half of the people who will suffer from a mental disorder having their first episode before 18 years of age.^[30]^ There is some evidence that receiving mental health information at school can result in reductions in stigmatizing attitudes.^[22,31]^ Further research, intervention and activism are needed in the field of mental health in Arab countries to improve the awareness of mental health problems.^[32]^

Despite the need for such program especially in our region, where there is more stigma attitude, no studies or few have been conducted shed the light on this important area of research.

The proposed research will assist in improving mental health literacy on depression following an educational intervention program. Strengths include that the program will be conducted by students’ teachers at their schools, mimicking the natural sitting, which will lead to outcomes that can be generalized. If positive trends result from the present trial, they will support the development of further longitudinal work on a larger scale, for combating the stigma associated with depression, which is urgently needed in this field.

Limitations include the fact that the intervention will be conducted by 2 independent teachers, 1 from each school, but effect of this will be minimized by giving a short training for both teachers so that they would deliver identical programs.

## Acknowledgments

The authors would like to thank Professor Kathy Griffiths for permission to use and translate the D-Lit, DSS, and SSDS Questionnaires.^[33,34]^

## Author contributions

HD is chief investigator on the project. HD, MM, RS, and MB contributed to the development and implementation of the intervention. All authors contributed to the design of the study. All authors contributed to the drafting of this manuscript and all approved the final version of this manuscript for publication.

**Conceptualization:** Hussain Darraj, Mohamed Salih Mahfouz, Rashad Al Sanosi, Mohammed Badedi, Abdullah Sabai.

**Methodology:** Hussain Darraj, Rashad Al Sanosi, Mohammed Badedi.

**Project administration:** Mohamed Salih Mahfouz.

**Supervision:** Mohamed Salih Mahfouz.

**Writing – original draft:** Hussain Darraj, Mohammed Badedi, Abdullah Sabai.

**Writing – review and editing:** Hussain Darraj, Mohamed Salih Mahfouz, Rashad Al Sanosi, Abdullah Sabai.

## Supplementary Material

Supplemental Digital Content
