# Supplementary material for: The effects of an educational program on depression literacy and stigma among students of secondary schools in Jazan city, 2016: A cluster-randomized controlled trial study protocol
Source: Medicine (Baltimore). 2018 May 4;97(18):e9433. doi: 10.1097/MD.0000000000009433 (PMC6392773; doi:10.1097/MD.0000000000009433)

**Figure 1**

**Recruitment of samples, flow of students through trial recruitment and randomization to control and intervention groups.**


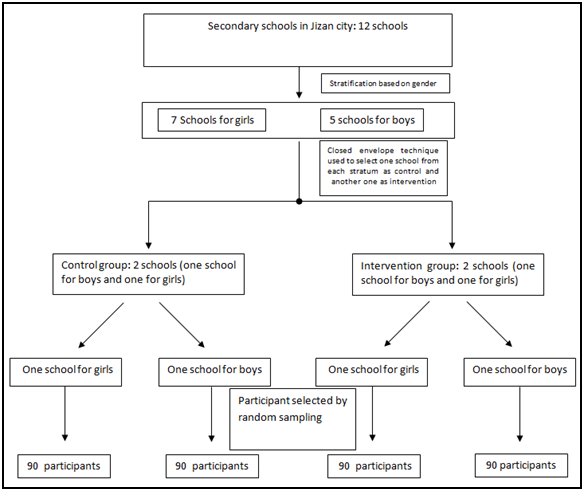

Supplement: Supplemental Digital Content [file medi-97-e9433-s001.docx]
